# Associations of Physical Activity, Vegetarian Status, and Sleep Duration with Psychological Distress in Peruvian Adults: Model-Based Indirect Associations via Dietary Self-Efficacy

**DOI:** 10.3390/nu18121907

**Published:** 2026-06-12

**Authors:** Jacksaint Saintila, Ramos Alfonso Paredes-Aguirre, Marilú Elena Barreto Espinoza, Antonio Serpa-Barrientos, Juan Marcelo Zanga Céspedes

**Affiliations:** 1 Research Group on Nutritional Psychology and Public Health, Universidad Peruana Unión, Lima 15464, Peru; 2 Escuela de Posgrado, Unidad de Posgrado de Salud, Universidad Peruana Unión, Lima 15464, Peru; alfonso.paredes@upeu.edu.pe; 3 Escuela de Psicología, Departamento Académico de Psicología, Universidad Nacional de Tumbes, Tumbes 24001, Peru; mbarretoe@untumbes.edu.pe; 4 Departamento de Psicología, Universidad Nacional Mayor de San Marcos, Lima 15081, Peru; aserpab@unmsm.edu.pe; 5 Facultad de Teología, Universidad Peruana Unión, Lima 15464, Peru; marcelozanga@upeu.edu.pe

**Keywords:** psychological distress, dietary self-efficacy, vegetarian status, physical activity, sleep duration, adults

## Abstract

**Background:** Psychological distress (PD) has been associated with lifestyle behaviors, including physical activity, sleep duration, and diet-related practices. Dietary self-efficacy may represent a self-regulatory correlate in these relationships; however, evidence from population studies remains limited, particularly in Peruvian adults. Objective: To examine whether dietary self-efficacy is statistically associated with the links between physical activity, sleep duration, vegetarian status, and PD in a sample of Peruvian adults. **Methods:** A cross-sectional study was conducted in 684 Peruvian adults. Structural equation modeling (SEM) was used to evaluate the proposed associations, adjusting for age, sex, educational level, marital status, residence, and BMI. **Results:** The SEM showed acceptable fit, with RMSEA and SRMR within recommended ranges and CFI/TLI slightly below 0.90 (CFI = 0.898, TLI = 0.886, RMSEA = 0.039, SRMR = 0.047). Lower dietary self-efficacy was associated with higher PD (β = 0.283, *p* < 0.001). Physical activity showed an indirect statistical association with lower PD via dietary self-efficacy (β = −0.043, *p* < 0.001) and a significant total association with PD (β = −0.085, *p* = 0.018). Sleep duration showed a curvilinear (U-shaped) association with PD (linear β = −0.122, *p* = 0.001; quadratic β = 0.124, *p* < 0.001), but not via dietary self-efficacy. Vegetarian status was not directly associated with PD (β = 0.002, *p* = 0.956), and its indirect statistical association via dietary self-efficacy did not reach conventional significance (β = 0.022, *p* = 0.070). The model explained 14.1% of the variance in dietary self-efficacy and 19.3% of the variance in PD. **Conclusions:** Lower dietary self-efficacy was associated with higher PD and captured a model-based indirect statistical association between physical activity and PD. Given the cross-sectional design, these findings should be interpreted as correlational and warrant confirmation in longitudinal or experimental studies.

## 1. Introduction

Psychological distress (PD) is a core component of mental and physical health, as it is associated with poorer quality of life, impaired social functioning, and an increased risk of mental disorders [1]. In Peru, research has documented substantial PD and depressive symptoms among adults, particularly during and after the COVID-19 pandemic and other crises [2,3]. For example, in a sample from Peru and Ecuador, adults reported moderate to high levels of eudaimonic well-being (self-acceptance, positive relations, personal growth, and purpose in life), although these levels were lower than those observed in Spain and Mexico and higher than those reported in Venezuela [3]. Among adults, mental health outcomes are influenced not only by individual and contextual factors but also by modifiable lifestyle behaviors, notably physical activity, sleep duration, and diet [4]. These behaviors are recognized as important determinants of mental health, prompting growing interest in understanding their interrelationships and their associations with PD.

Dietary self-efficacy is defined as the perceived ability to regulate eating behavior in the face of risk situations, such as exposure to highly palatable foods, social pressures, or negative emotional states [5]. From the perspective of Social Cognitive Theory, developed by Bandura [6], self-efficacy represents a key self-regulatory belief, as it influences behavior selection, the effort invested, and persistence in the face of obstacles [7]. Available evidence indicates that dietary self-efficacy is associated with better adherence to healthier dietary practices [8]. However, a study conducted among Turkish adults reported that higher dietary self-efficacy was associated with a lower overall dietary pattern score [9].

One of the most widely used instruments for assessing dietary self-efficacy is the Dietary Self-Efficacy Scale (DIET-SE), which conceptualizes dietary self-efficacy as a multidimensional construct composed of three first-order domains: (1) high-calorie food temptations (HCFT), (2) social and internal factors (SIF), and (3) negative emotional events (NEE) [10]. However, despite the theoretical relevance of dietary self-efficacy as a self-regulatory resource [6,7], evidence examining its potential role in the associations between lifestyle behaviors—such as physical activity, sleep duration, and vegetarian status—and psychological distress remains limited.

Regular physical activity has been associated with lower levels of depressive and anxiety symptoms and lower PD [11]. However, this relationship is not always direct and may depend on intermediate psychosocial processes, such as self-efficacy [12]. Lifestyle behaviors tend to co-occur and may share self-regulatory processes. In this context, dietary self-efficacy may reflect a broader self-regulation resource that is also related to maintaining physical activity routines and sleep-related behaviors, making it a relevant construct to examine within an integrated model. Similarly, sleep duration and quality have shown consistent associations with mood states and PD [13,14]. Like physical activity, sleep does not operate in isolation but rather interacts with other health behaviors, particularly eating behaviors or overall diet quality [15]. Accordingly, dietary self-efficacy is conceptually consistent with these models, although its potential role in the associations between physical activity, sleep duration, and psychological distress has not been well characterized.

Diet has also been extensively studied in relation to mental health, although findings have been heterogeneous. Plant-based dietary patterns, including vegetarian diets, have shown inconsistent associations with mental health outcomes. While some studies report better mood states and a lower prevalence of depressive symptoms [16], others have found no significant differences or have even described greater vulnerability, including higher depression scores compared with non-vegetarians [17,18]. More recent evidence from large cross-sectional and cohort studies using plant-based diet quality indices suggests that healthy plant-based diets—characterized by higher intakes of whole grains, fruits, vegetables, legumes, and nuts—are associated with lower odds of depression, anxiety, and PD, whereas lower-quality plant-based diets rich in refined grains, sweets, and fried foods are associated with higher psychological symptomatology [19,20]. In the present study, diet was operationalized as vegetarian status (vegetarian vs. non-vegetarian), which captures diet type rather than comprehensive dietary patterns derived from detailed dietary assessments.

To the best of our knowledge, few studies have examined whether dietary self-efficacy is associated with physical activity, sleep duration, vegetarian status, and psychological distress within an integrated model, particularly in Latin American adult populations. Addressing this gap may contribute to a more integrated understanding of lifestyle behaviors and mental health by evaluating a hypothesized model in which dietary self-efficacy is directly associated with PD and captures indirect associations between lifestyle behaviors and distress.

Therefore, the present study aimed to evaluate the hypothesized structural relations among lifestyle behaviors and PD by estimating a SEM in which dietary self-efficacy functions as a self-regulatory correlate linking physical activity, sleep duration (including potential non-linear effects), and vegetarian status with PD in Peruvian adults. Based on the theoretical framework and prior evidence, we formulated the following hypotheses:

**H1.**  *Lower dietary self-efficacy would be associated with higher PD.*

**H2.**  *Physical activity would show an indirect statistical association with PD via dietary self-efficacy.*

**H3.**  *Vegetarian status (vegetarian vs. non-vegetarian) would show an indirect statistical association with PD via dietary self-efficacy.*

**H4.**  *Sleep duration would show a U-shaped association with PD; dietary self-efficacy was hypothesized to be statistically related to this association.*

## 2. Materials and Methods

### 2.1. Design and Participants

A cross-sectional study was conducted among Peruvian adults. The study population consisted of individuals recruited through religious, community, and educational networks. Participants were drawn from different regions of the country (coastal, highland, and jungle areas), with a minimal proportion of foreign residents.

Participants were recruited using non-probabilistic convenience sampling through community activities, educational settings, and university spaces. Inclusion criteria were being 18 years of age or older. Questionnaires with inconsistent responses were excluded, defined as: (a) logical contradictions between key items, (b) invariant or patterned responding suggestive of inattention, or (c) missing responses exceeding 10% of the Dietary Self-Efficacy Scale (DIET-SE) or General Health Questionnaire (GHQ-12) items.

The adequacy of the sample size for the structural equation model was evaluated using the *semPower* package in R (version 4.4.1), applying an RMSEA-based approach. The analysis assumed RMSEA = 0.05, a significance level of α = 0.05, statistical power of 0.80, and the model degrees of freedom (df = 292) as specified in the SEM. Under these assumptions, the minimum required sample size was N = 91. The final analytic sample (N = 684) exceeded this requirement by 593 participants (≈7.5-fold), providing adequate power for model estimation.

### 2.2. Procedure

The questionnaires were administered in person by two trained research assistants, following a standardized administration protocol. To minimize potential bias, interviewers were not informed of the specific study hypotheses and were limited to providing general instructions on how to complete the instruments, without offering additional interpretations. Data collection was conducted individually and under conditions that ensured participants’ privacy. Participation was voluntary and anonymous, and all participants provided written informed consent prior to completing the questionnaires. Subsequently, the questionnaires were reviewed to verify response consistency, and those that did not meet the established quality criteria were excluded.

### 2.3. Instruments and Variables

***Dietary self-efficacy.*** The Dietary Self-Efficacy Scale (DIET-SE) [10] was used to assess perceived ability to regulate eating behavior in challenging situations and to resist unplanned eating. The scale comprises 11 items grouped into three dimensions: high-calorie food temptations (HCFT; 4 items; e.g., “When I am faced with foods that I like very much”), social and internal factors (SIF; 4 items; e.g., “When other people encourage me to eat excessively”), and negative emotional events (NEE; 3 items; e.g., “When I feel sad or anxious”) [21]. Items are rated on an ordinal response scale (0–4). In line with prior applications of the DIET-SE, higher scores indicate lower dietary self-efficacy (i.e., greater difficulty controlling food intake under risk situations). In the present sample, the DIET-SE showed excellent internal consistency (Cronbach’s α = 0.93; composite reliability = 0.93), supporting its adequacy for assessing dietary self-efficacy in this population.

***Psychological distress***. PD was assessed using the 12-item General Health Questionnaire (GHQ-12), a widely used screening measure of recent mental health problems over the past few weeks. The questionnaire includes six positively worded items (items 1, 3, 4, 7, 8, and 12) and six negatively worded items (items 2, 5, 6, 9, 10, and 11). Responses are recorded on a four-point Likert scale (0–1–2–3) reflecting the frequency or severity of symptoms or perceptions during the past weeks (e.g., “Have your worries made you lose much sleep?”). Total scores range from 0 to 36, with higher scores indicating greater PD (worse mental health). A cutoff score of ≥11 has been suggested to indicate poor mental health [22]. Responses were treated as ordinal (0–3). In the analyzed sample, the GHQ-12 showed excellent internal consistency (Cronbach’s α = 0.91; composite reliability = 0.92).

***Physical activity***. Physical activity was assessed using a self-report item that asked about the weekly frequency of leisure-time exercise. Responses were grouped into four categories: less than once per week, 1–2 times per week, 3–4 times per week, and five or more times per week [23,24]. This classification was adopted due to its frequent use in population-based studies and its consistency with international recommendations, which indicate that adults should engage in moderate-to-vigorous physical activity at least five times per week to obtain relevant health benefits [25].

***Sleep duration***. Sleep duration was assessed based on the average number of hours of sleep per day self-reported by participants. For descriptive purposes, this variable was categorized into three groups: less than 7 h per day, between 7 and 9 h per day, and more than 9 h per day, in accordance with ranges commonly used in research on sleep habits in adult populations [26].

***Vegetarian status***. Vegetarian status was assessed using a direct question about participants’ usual eating regimen. Based on responses, participants were classified as vegetarian or non-vegetarian. Participants were classified as vegetarian if they reported following a vegan or lacto-ovo-vegetarian diet, defined by the exclusion of meat and, in the case of vegans, all animal-derived products. Participants were classified as non-vegetarian if they reported regular consumption of red meat, poultry, fish, and eggs more than once per week [27].

### 2.4. Ethical Considerations

The study protocol was reviewed and approved by the Institutional Ethics Committee of Universidad Peruana Unión on 6 February 2024. All procedures were conducted in accordance with the principles of the Declaration of Helsinki. Participants were informed about the study objectives, the confidentiality of the information, and their right to withdraw at any time without consequences.

### 2.5. Statistical Analysis

Statistical analyses were conducted using R (version 4.4.1). Descriptive statistics included frequencies and percentages for categorical variables and means with standard deviations for continuous variables. Missing data were minimal (overall proportion = 0.0079%; maximum per variable = 0.1304%) and were handled through listwise deletion; subsequent analyses were estimated with the Weighted Least Squares Mean and Variance adjusted (WLSMV) estimator.

Internal consistency was evaluated using Cronbach’s alpha (α) and composite reliability (CR), and convergent validity was assessed through the average variance extracted (AVE). Bivariate associations were examined using Pearson correlations; Spearman correlations were additionally computed as a sensitivity analysis due to ordinal predictors, yielding a similar pattern of results. Multicollinearity was evaluated using variance inflation factor (VIF < 5) and tolerance (>0.20).

The factorial structure of dietary self-efficacy was examined using confirmatory factor analysis (CFA) specifying a second-order model with three first-order factors. Given the ordinal response format of the items, all indicators were specified as ordered and estimated.

A SEM was subsequently estimated to examine direct and model-based indirect statistical associations between physical activity, sleep duration (modeled using centered linear and quadratic terms), vegetarian status, and psychological distress via dietary self-efficacy. The SEM was adjusted for age, sex, educational level, marital status, residence, and BMI.

Model fit was evaluated using CFI, TLI, RMSEA (90% CI), and SRMR, with CFI/TLI ≥ 0.90 and RMSEA/SRMR ≤ 0.08 indicating acceptable fit. Direct, indirect, and total associations were reported as standardized coefficients (β). Indirect and total relations were defined within the SEM using model constraints and evaluated using 95% confidence intervals derived from robust standard errors (delta method). Explained variance (R^2^) for endogenous variables was reported. Statistical significance was set at *p* < 0.05. Physical activity was entered as an ordered predictor (1–4) reflecting increasing weekly frequency, capturing a monotonic trend across categories. Vegetarian status was entered as a binary observed predictor (1 = vegetarian, 2 = non-vegetarian).

## 3. Results

The sample comprised 684 participants with a mean age of 35.73 years (SD = 14.31), of whom 60.8% were female. Most participants were from the jungle region (43.0%), followed by the coast (30.6%) and highlands (25.9%), and the majority resided in urban areas (58.5%). In terms of marital status, 58.6% were married, and 54.2% had university-level education. Most participants were non-vegetarian (72.2%). Regarding lifestyle factors, the most common frequency of physical activity was 1–2 times per week (48.2%), while 65.8% reported sleeping 7–9 h per day; the mean sleep duration was 6.94 h (SD = 1.12) (see Table 1).

Table 2 presents the descriptive statistics of the study variables. Dietary self-efficacy had a mean of 18.06 (SD = 8.80) and an approximately symmetric distribution. PD showed a mean of 10.62 (SD = 6.32), with slight positive skewness, indicating a greater concentration of scores at lower levels and a tail toward higher distress values. Sleep duration averaged 6.94 h/day (SD = 1.11), showing low dispersion and an approximately normal distribution.

Table 3 presents the bivariate correlations, reliability indices, and collinearity diagnostics of the study variables. DIET-SE was positively correlated with PD (r = 0.275), indicating that higher DIET-SE scores—reflecting lower dietary self-efficacy—are associated with higher PD. Physical activity showed negative correlations with DIET-SE (r = −0.161) and PD (r = −0.103), suggesting that higher physical activity is associated with lower DIET-SE scores (i.e., higher dietary self-efficacy) and lower distress. Vegetarian status showed positive, low-magnitude correlations with DIET-SE (r = 0.136) and PD (r = 0.039). Given the coding of the variable (higher values = non-vegetarian), these correlations suggest that non-vegetarians tended to report lower dietary self-efficacy and slightly higher psychological distress than vegetarians.

Regarding reliability, both dietary self-efficacy and psychological distress demonstrated excellent internal consistency, with α values above 0.90 and high CR (≥0.91). The AVE was adequate for dietary self-efficacy (0.821) and acceptable for psychological distress (0.478). Regarding collinearity assumptions, VIF values ranged from 1.011 to 1.025, and tolerance values were above 0.97 for all predictor variables, indicating the absence of multicollinearity issues and supporting the stability of the structural model estimates.

### 3.1. Measurement Model (CFA)

Prior to the structural model, a second-order CFA was estimated. The measurement model showed acceptable-to-good fit: CFI = 0.974, TLI = 0.970, RMSEA = 0.070 (90% CI: 0.065–0.074), and SRMR = 0.062. Standardized factor loadings ranged from 0.565 to 0.782 for the DIET-SE items and from 0.538 to 0.839 for the PD items, and all loadings were statistically significant (*p* < 0.001). Second-order loadings of HCFT (β = 0.813), SIF (β = 0.951), and NEE (β = 0.940) onto dietary self-efficacy supported the hierarchical structure of the DIET-SE construct, indicating that the three domains reflect a common higher-order self-regulatory construct rather than independent dimensions.

### 3.2. Overall Fit of the Structural Model

The proposed SEM showed an acceptable overall fit to the empirical data. The goodness-of-fit indices indicated satisfactory model performance for RMSEA and SRMR, while CFI and TLI were slightly below the conventional 0.90 threshold: scaled χ^2^ = 937.04 with 436 degrees of freedom, CFI = 0.898, TLI = 0.886, RMSEA = 0.039 (90% CI: 0.035–0.042), and SRMR = 0.047.

**Figure 1:**
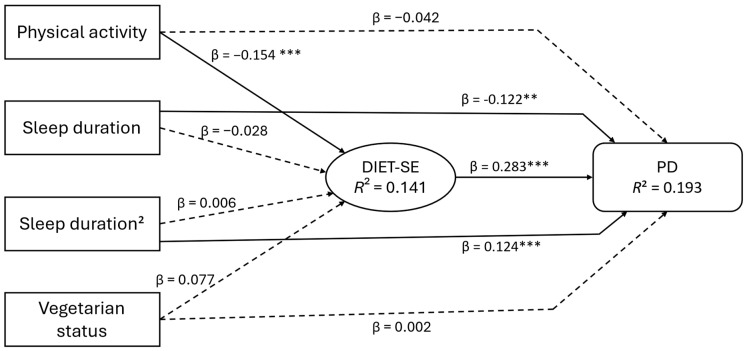
Structural equation model of the associations among physical activity, sleep duration, vegetarian status, dietary self-efficacy, and psychological distress. *Note.* Standardized coefficients (β) are presented. Solid lines indicate statistically significant associations, whereas dashed lines indicate non-significant associations. ** *p* < 0.01, *** *p* < 0.001. DIET-SE = Dietary Self-Efficacy; PD = Psychological Distress. Higher DIET-SE scores indicate lower dietary self-efficacy, and higher PD scores indicate greater psychological distress. The model was adjusted for age, sex, educational level, marital status, residence, and body mass index (BMI). R^2^ values represent the proportion of explained variance in the endogenous variables.

Table 4 presents the direct, indirect, and total associations of the SEM, and the graphical representation is shown in Figure 1. Physical activity was inversely associated with DIET-SE (β = −0.154, *p* < 0.001), whereas sleep duration (linear and quadratic terms) was not associated with DIET-SE (*p* ≥ 0.453). Vegetarian status showed a small positive association with DIET-SE that did not reach conventional significance (β = 0.077, *p* = 0.063). DIET-SE showed a direct, positive association with PD (β = 0.283, *p* < 0.001). Sleep duration was directly associated with PD in a curvilinear pattern (linear β = −0.122, *p* = 0.001; quadratic β = 0.124, *p* < 0.001), whereas physical activity (β = −0.042, *p* = 0.242) and vegetarian status (β = 0.002, *p* = 0.956) were not directly associated with PD. Regarding indirect associations via DIET-SE, physical activity showed a significant association with PD (β = −0.043, *p* < 0.001), while the corresponding associations for sleep duration and vegetarian status were not statistically significant (*p* ≥ 0.070). Total associations indicated significant links of physical activity (β = −0.085, *p* = 0.018) and sleep duration (linear β = −0.130, *p* < 0.001; quadratic β = 0.125, *p* < 0.001) with PD, whereas the total association for vegetarian status was not significant (β = 0.024, *p* = 0.563).

## 4. Discussion

The fit indices of the structural equation model indicated satisfactory overall performance. RMSEA and SRMR indicated good model fit, whereas CFI and TLI were slightly below the conventional 0.90 threshold. These results support the interpretation of the estimated structural paths and the model-based indirect associations involving dietary self-efficacy within the specified SEM; however, given the cross-sectional design, the findings should be interpreted as associational rather than evidence of temporal ordering or causal mediation.

In relation to H1, DIET-SE showed a direct, positive association with PD. This finding suggests that lower perceived ability to regulate eating behavior is associated with higher PD, whereas higher dietary self-efficacy is linked to lower distress. This interpretation is consistent with previous studies reporting that a stronger perceived sense of control over eating behavior and related self-regulatory beliefs are associated with more favorable mental health indicators, including higher life satisfaction and positive affective states [28,29,30]. These results can be interpreted in light of self-regulation and self-efficacy frameworks, which posit that beliefs in one’s ability to manage health-related behaviors contribute to a greater sense of personal mastery, reduced PD, and better emotional adjustment [31,32]. Accordingly, dietary self-efficacy may be viewed not only as confidence in resisting dietary temptations but also as a psychological resource that supports perceived control in everyday life [31].

Physical activity was not directly associated with PD; however, it showed a significant indirect association via dietary self-efficacy. Considering the DIET-SE scoring direction, more frequent physical activity was associated with higher dietary self-efficacy, which in turn was associated with lower distress. This is consistent with research indicating that physical activity may relate to eating behaviors and mental health outcomes through psychosocial processes, including self-regulation and self-efficacy. In particular, Annesi [33] reported that changes in physical activity are associated with improvements in diet and body weight through a sequence of psychosocial factors, including reductions in anxiety and depression, decreases in emotional eating, and strengthening of dietary self-efficacy. Similarly, another study [11] found that increases in physical activity are linked to more favorable mood-related outcomes and reduced emotional eating particularly when accompanied by self-regulatory skills and increases in self-efficacy, rather than through direct effects of physical activity per se. Taken together, these findings support the interpretation that physical activity may be associated with lower PD insofar as it co-occurs with stronger self-regulatory beliefs that extend to the dietary domain.

Results related to H3 did not provide strong support for an association between vegetarian status and psychological distress in this sample. Vegetarian status was neither directly associated with PD nor did it show a statistically significant indirect association via dietary self-efficacy. Although the association approached conventional significance thresholds, its magnitude was small and should be interpreted cautiously.

This suggests that vegetarian status alone is not a proxy for diet quality, adherence, or the psychological meanings attached to dietary choices. In practice, individuals classified as “vegetarian” may follow markedly different dietary profiles (e.g., minimally processed plant-based foods vs. refined carbohydrates and ultra-processed products), and this heterogeneity may obscure associations with psychological distress. Prior evidence on vegetarian dietary patterns and mental health outcomes has been mixed, with some studies reporting better mood states and lower depressive/anxiety symptoms among vegetarians, whereas others find no differences or even higher vulnerability to psychological symptoms [34,35,36]. A plausible explanation for these discrepancies is that mental health correlates depend less on vegetarian status per se and more on diet quality (healthy vs. unhealthy plant-based patterns), degree of adherence, and contextual variables such as motivations for adopting the diet and social support [34,35,36].

In the same way, it is also possible that dietary self-efficacy plays a more salient role in shaping mental health outcomes when it is directly tied to concrete dietary behaviors and perceived control in daily eating situations. While previous studies have shown that self-efficacy is associated with greater adherence to plant-based diets and intentions to maintain such practices [9,37], our results indicate that these self-regulatory beliefs did not translate into a robust indirect association between vegetarian status and PD within the present model. Future studies should therefore incorporate more detailed dietary assessments (e.g., plant-based diet quality indices, frequency-based intake measures, or healthy/unhealthy plant-based diet scores), as well as measures of adherence, duration of dietary practice, and motivations (health vs. ethical vs. religious reasons). These refinements may clarify whether any association between plant-based eating and psychological distress is contingent on diet quality and self-regulatory resources.

When evaluating H4, the results supported the expected U-shaped association between sleep duration and PD, but they did not support an indirect statistical association through DIET-SE. Specifically, the significant linear and quadratic sleep parameters indicated that departures from average sleep duration were associated with higher distress. However, sleep duration was not significantly associated with DIET-SE, and the corresponding indirect statistical association was not significant. Empirical evidence specifically examining dietary self-efficacy in relation to the association between sleep duration and psychological distress remains limited. Most available studies have focused on direct associations between sleep duration and indicators such as depressive symptoms and subjective well-being, without explicitly testing diet-related self-regulatory processes [13,14]. Recent population-based studies further suggest that unhealthy sleep duration is associated with a higher risk of depressive symptoms, particularly when co-occurring with poor diet quality, pointing to the clustering of lifestyle behaviors rather than a clearly established explanatory process [13,14,38]. Therefore, the present findings contribute to the literature by indicating that dietary self-efficacy was directly associated with psychological distress and was involved in the statistical association between physical activity and distress within the proposed SEM, whereas the sleep–distress association appeared to be primarily direct and independent of dietary self-efficacy in this sample.

The results are broadly consistent with the proposed model by showing that dietary self-efficacy is a central construct in the associations between lifestyle behaviors (physical activity, sleep duration, and vegetarian status) and PD. Although the explained variance is modest (R^2^ = 0.141 for dietary self-efficacy and R^2^ = 0.193 for psychological distress), these values are consistent with behavioral health research, where psychological distress reflects the interplay of multiple biological, psychological, and social determinants. Importantly, the present study advances the literature by jointly modeling physical activity, sleep duration (including a quadratic term), and vegetarian status within a single SEM, while accounting for key sociodemographic covariates and examining their associations with psychological distress. Nevertheless, given the cross-sectional design, the observed associations should be interpreted as correlational and warrant confirmation in longitudinal and intervention studies.

### 4.1. Limitations and Future Lines of Research

The present study has several limitations. First, the cross-sectional design precludes establishing temporal ordering or causal relationships; therefore, the estimated direct and indirect effects should be interpreted as model-based associations rather than causal mediation. Longitudinal and intervention studies are needed to test directionality and change processes.

Second, participants were recruited via non-probabilistic convenience sampling, which limits generalizability. Moreover, a substantial proportion of respondents were drawn from religious communities that promote healthy lifestyle practices, raising the possibility of selection bias and context-specific patterns. Replication in more diverse and representative samples is warranted, including tests of whether community or cultural context moderates the observed associations.

Third, lifestyle behaviors were assessed by self-report, which is vulnerable to recall and social desirability bias. Because all variables were self-reported and collected concurrently, common method variance cannot be ruled out. In addition, diet was operationalized as vegetarian status, which does not capture diet quality, adherence, or motivations; thus, vegetarian status should not be interpreted as a proxy for healthy eating. Future research should incorporate more detailed dietary assessments (e.g., diet quality indices, FFQs or recalls), adherence indicators, and motivations, including distinctions between healthy vs. unhealthy plant-based patterns.

Fourth, despite adjustment for key covariates, other relevant determinants were not measured (e.g., perceived stress, emotional eating, emotion regulation, socioeconomic status, chronic conditions, and social support), which may contribute to the modest explained variance. Future work should incorporate these constructs and evaluate alternative specifications, including potential interactions among lifestyle behaviors and nonlinear associations (particularly for sleep).

Finally, missing data and response quality may have influenced estimates; future studies should report missingness patterns and prespecified criteria for inconsistent responses, and consider robust missing-data approaches (e.g., multiple imputation) when appropriate. Measurement and structural invariance across relevant subgroups (e.g., sex, age, SES) should also be examined to support comparability.

### 4.2. Practical and Public Health Implications

The findings have relevant implications for clinical practice and public health strategies aimed at promoting mental health in adults. The results suggest that dietary self-efficacy is associated with psychological distress and may represent an important self-regulatory factor within broader lifestyle–mental health relationships. Accordingly, interventions focused exclusively on behavioral targets may benefit from incorporating components that strengthen individuals’ confidence in managing eating behaviors across challenging situations.

From a practical perspective, lifestyle promotion programs could incorporate psychoeducational and self-regulation strategies, such as goal setting, management of dietary temptations, identification of emotional eating triggers, and development of coping skills. These approaches may support greater dietary self-efficacy and may be associated with lower psychological distress. From a public health perspective, the findings support integrated approaches that jointly address lifestyle behaviors and psychological well-being. In particular, physical activity was associated with dietary self-efficacy, which in turn was associated with psychological distress, suggesting that self-regulatory processes may be relevant when designing health promotion programs. Finally, dietary self-efficacy may represent a potentially scalable target for mental health promotion. However, given the cross-sectional design, longitudinal and intervention studies are needed to determine whether strengthening dietary self-efficacy is prospectively associated with improvements in PD.

## 5. Conclusions

Dietary self-efficacy was directly associated with psychological distress in this sample of Peruvian adults, with lower perceived ability to regulate eating behavior being associated with greater distress. Within the proposed structural equation model, dietary self-efficacy was involved in the statistical association between physical activity and psychological distress, whereas no significant indirect associations were observed for sleep duration or vegetarian status. Sleep duration showed a significant U-shaped association with psychological distress, indicating that both shorter and longer sleep durations were associated with greater distress. Vegetarian status was not significantly associated with psychological distress. Given the cross-sectional design, these findings should be interpreted as model-based associations rather than evidence of temporal ordering or causal processes. Future longitudinal and intervention studies are needed to clarify the role of dietary self-efficacy within lifestyle–mental health relationships.

## Figures and Tables

**Table 1:** Sociodemographic and lifestyle characteristics of the sample (n = 684).

Variable	n	%
**Age (years)**		
Mean ± SD	—	35.73 ± 14.31
**Sex**		
Female	416	60.8
Male	268	39.2
**Region**		
Coast	209	30.6
Jungle	294	43.0
Highlands	177	25.9
Foreign	4	0.6
**Place of residence**		
Urban	400	58.5
Rural	284	41.5
**Marital status**		
Single	283	41.4
Married	401	58.6
**Educational level**		
Basic	212	31.0
Technical	101	14.8
University	371	54.2
**Vegetarian status**		
Vegetarian	190	27.8
Non-vegetarian	494	72.2
**Physical activity**		
Never	104	15.2
1–2 times/week	330	48.2
3–4 times/week	120	17.5
≥5 times/week	130	19.0
**Sleep duration (categorical)**		
<7 h/day	220	32.2
7–9 h/day	450	65.8
>9 h/day	14	2.0
**Sleep duration (hours)**		
Mean ± SD	—	6.94 ± 1.12

***Note.*** M = mean; SD = standard deviation; n = number of participants. Values are presented as mean ± standard deviation and as absolute frequency and percentage, as appropriate.

**Table 2:** Descriptive statistics of the study variables.

Variable	M	SD	CV	Skewness	Kurtosis	Min	Max
DIET-SE	18.06	8.80	0.49	−0.05	−0.44	0	42
PD	10.62	6.32	0.60	0.63	0.07	0	36
PA	2.37	0.94	0.40	0.47	−0.72	1	4
SD	6.94	1.11	0.16	−0.02	1.75	3	12
VS	1.29	0.46	0.35	0.91	−1.18	1	2

***Note.*** Mean (M), standard deviation (SD), coefficient of variation (CV), minimum (Min), maximum (Max). DIET-SE = Dietary Self-Efficacy; PD = Psychological distress; PA = Physical Activity; SD = Sleep Duration; VS = Vegetarian Status. Means and standard deviations for PA and VS are reported for descriptive purposes only, as these variables were measured using ordinal response categories rather than continuous scales.

**Table 3:** Correlation analysis, reliability, and collinearity diagnostics of study variables.

Variables	1	2	3	4	5	α	CR	AVE	Tolerance	VIF
1. DIET-SE	—					0.93	0.932	0.821	—	—
2. PD	0.275	—				0.91	0.915	0.478	—	—
3. PA	−0.161	−0.103	—			—	—	—	0.989	1.011
4. SD	−0.023	−0.104	0.099	—		—	—	—	0.976	1.025
5. VS	0.136	0.039	−0.022	0.119	—	—	—	—	0.985	1.015

***Note.*** Pearson correlation coefficients are reported. Cronbach’s alpha (α) was estimated from observed indicators. CR = composite reliability; AVE = average variance extracted; VIF = variance inflation factor. Although the AVE for PD was slightly below the recommended threshold of 0.50, its high composite reliability (CR > 0.70) supports adequate convergent validity. No multicollinearity issues were detected (VIF < 5; tolerance > 0.20). DIET-SE = Dietary Self-Efficacy; PD = Psychological distress; PA = Physical Activity; SD = Sleep Duration; VS = Vegetarian Status.

**Table 4:** Direct, indirect, and total statistical associations in the structural equation model.

Association	β (Std.all)	SE	z	*p*	95% CI
**Direct associations with DIET-SE**					
Physical activity → DIET-SE	−0.154	0.022	−4.17	<0.001	[−0.197, −0.111]
Sleep duration (linear) → DIET-SE	−0.028	0.019	−0.75	0.453	[−0.065, 0.009]
Sleep duration^2^ (quadratic) → DIET-SE	0.006	0.007	0.21	0.833	[−0.008, 0.020]
Vegetarian status → DIET-SE	0.077	0.051	1.86	0.063	[−0.023, 0.177]
**Direct associations with PD**					
DIET-SE → PD	0.283	0.039	7.10	<0.001	[0.207, 0.359]
Physical activity → PD	−0.042	0.021	−1.17	0.242	[−0.083, 0.000]
Sleep duration (linear) → PD	−0.122	0.018	−3.41	0.001	[−0.157, −0.087]
Sleep duration^2^ (quadratic) → PD	0.124	0.007	3.90	<0.001	[0.110, 0.138]
Vegetarian status → PD	0.002	0.049	0.06	0.956	[−0.094, 0.098]
**Indirect statistical associations via DIET-SE**					
Physical activity → DIET-SE → PD	−0.043	0.007	−3.65	<0.001	[−0.057, −0.029]
Sleep duration (linear) → DIET-SE → PD	−0.008	0.005	−0.74	0.460	[−0.018, 0.002]
Sleep duration^2^ → DIET-SE → PD	0.002	0.002	0.21	0.833	[−0.002, 0.006]
Vegetarian status → DIET-SE → PD	0.022	0.015	1.81	0.070	[−0.007, 0.051]
**Total statistical associations**					
Physical activity → PD	−0.085	0.021	−2.38	0.018	[−0.091, −0.009]
Sleep (linear) → PD	−0.130	0.018	−3.50	<0.001	[−0.100, −0.028]
Sleep^2^ → PD	0.125	0.008	3.81	<0.001	[0.014, 0.043]
Vegetarian status → PD	0.024	0.050	0.58	0.563	[−0.069, 0.128]
**Explained variance**					
DIET-SE	R^2^ = 0.141				
PD	R^2^ = 0.193				

***Note.*** DIET-SE = Dietary Self-Efficacy; PD = Psychological Distress; CI = Confidence Interval; SE = Standard Error; R^2^ = Explained Variance. Sleep duration^2^ represents the quadratic term for centered sleep duration. β values are standardized coefficients (Std.all). The model was adjusted for age, sex, educational level, marital status, residence, and BMI. Higher DIET-SE scores indicate lower dietary self-efficacy, and higher PD scores indicate greater psychological distress. Indirect and total associations were estimated using model constraints, and 95% CIs were obtained using the delta method.

## Data Availability

The datasets generated and/or analyzed during the current study are available from the corresponding author upon reasonable request due to privacy and ethical restrictions regarding participant confidentiality.

## Abbreviations

The following abbreviations are used in this manuscript:

AVE	Average Variance Extracted
CFA	Confirmatory Factor Analysis
CFI	Comparative Fit Index
CI	Confidence Interval
CR	Composite Reliability
DIET-SE	Dietary Self-Efficacy
GHQ-12	General Health Questionnaire-12
HCFT	High-Calorie Food Temptations
NEE	Negative Emotional Events
PA	Physical Activity
PD	Psychological Distress
RMSEA	Root Mean Square Error of Approximation
SD	Sleep Duration
SEM	Structural Equation Modeling
SIF	Social and Internal Factors
SRMR	Standardized Root Mean Square Residual
TLI	Tucker–Lewis Index
VIF	Variance Inflation Factor
VS	Vegetarian Status
